# Effectiveness of Text-Only Cigarette Health Warnings in Japan: Findings from the 2018 International Tobacco Control (ITC) Japan Survey

**DOI:** 10.3390/ijerph17030952

**Published:** 2020-02-04

**Authors:** Janet Chung-Hall, Geoffrey T. Fong, Gang Meng, Mi Yan, Takahiro Tabuchi, Itsuro Yoshimi, Yumiko Mochizuki, Lorraine V. Craig, Janine Ouimet, Anne C. K. Quah

**Affiliations:** 1 Department of Psychology, University of Waterloo, 200 University Ave W., Waterloo, ON N2L 3G1, Canada; gfong@uwaterloo.ca (G.T.F.); gmeng@uwaterloo.ca (G.M.); mi.yan@uwaterloo.ca (M.Y.); lvcraig@uwaterloo.ca (L.V.C.); j2ouimet@uwaterloo.ca (J.O.); ackquah@uwaterloo.ca (A.C.K.Q.); 2 Ontario Institute for Cancer Research, 661 University Ave, Suite 510, Toronto, ON M5G 0A3, Canada; 3 School of Public Health and Health Systems, University of Waterloo, 200 University Ave. W., Waterloo, ON N2L 3G1, Canada; 4 Cancer Control Center, Osaka International Cancer Institute, Chome-1-69 Otemae, Chuo Ward, Osaka 541-8567, Japan; tabuchitak@gmail.com; 5 Division of Tobacco Policy Research, National Cancer Center Japan, 5-1-1 Tsukiji, Chuo-ku, Tokyo 104-0045, Japan; iyoshimi@ncc.go.jp; 6 Japan Cancer Society, 13th Floor, Yurakucho Center Bldg. 2-5-1, Yurakucho, Chiyoda-ku, Tokyo 100-0006, Japan; mochizuki@jcancer.jp

**Keywords:** Japan, cigarette package warning labels, support, tobacco policy

## Abstract

Health warnings are an effective strategy for communicating the health harms of smoking, encouraging quitting, and preventing smoking initiation. This study examines the effectiveness of existing text-only health warnings, identifies key predictors of warning effectiveness, and assesses support for pictorial warnings in Japan. Data are from the 2018 International Tobacco Control (ITC) Japan Survey, a cohort survey of adult cigarette smokers (n = 3306), dual users of cigarettes and heated tobacco products (n = 555), and non-cigarette smokers (n = 823). Weighted multivariable logistic regression models were used to assess predictors of warning effectiveness and support for pictorial warnings. Overall, 15.6% of respondents noticed warnings, and 7.9% read or looked closely at warnings. Overall, 10.3% of smokers and dual users said the warnings stopped them from having a cigarette, and 7.2% avoided warnings. Overall, 27.5% of respondents said the warnings made them think about health risks of smoking, but only 2.7% of smokers and dual users said the warnings made them more likely to quit. Overall, 57.6% of respondents supported pictorial warnings. The weak effectiveness of Japan’s text-only warnings is consistent with that in other countries with similar warnings. There is majority support for pictorial warnings in Japan, although the level of support is lower than in other countries.

## 1. Introduction

Smoking is a major cause of premature mortality in Asia, accounting for approximately 2 million deaths in 2004 [1]. Japan is among the top ten countries with the largest smoking populations, where more than 20 million people smoke [2,3]. Smoking-related diseases such as cancer, heart disease, stroke, and chronic obstructive pulmonary disease are leading causes of premature death in Japan [4,5].

It is estimated that smoking kills more than 120,000 Japanese people each year, with the age-specific proportion of all-cause mortality for males being much higher in Japan (24.9%) than in other Asian countries with higher smoking rates such as China (12.9%) [6,7]. Although smoking prevalence is lower among Japanese females (9.3%) than males (26.6%) [2], a pooled meta-analysis of data from 20 prospective cohort studies across six Asian countries found that Japanese females are starting to smoke at a younger age in more recent birth cohorts compared to earlier cohorts, with an increase in cigarettes smoked per day in successive birth cohorts [8]. A population-based prospective study in Japan found that compared to never smokers, overall all-cause mortality was more than doubled for current smokers who started smoking before age 20 years, with life expectancy reduced by nearly 10 years among those who continued to smoke [9]. 

Prevention of non-communicable diseases caused by smoking is a critical challenge for population health in Japan, where smoking-related deaths are expected to rise substantially in the absence of effective policy interventions [10]. A recent modeling study that accounted for changes in smoking prevalence and the aging population in Japan estimated that the absolute number of cases and deaths from lung cancer alone will exceed 76,000 for men and 43,000 for women by 2035 [11].

A key objective of tobacco control is to increase awareness of the health harms of smoking, which has been shown to increase smokers’ quit intentions and quit attempts [12,13,14]. But very little is known about the level of awareness of the harms of smoking in Japan, a country that is the fifth largest consumer of cigarettes in the world [2]. A study of the Japanese general population found that cancer-causing viruses and bacterial infection (51%) were ranked as the top risk factor for cancer, followed by tobacco smoking (43%) [15]—when, in fact, tobacco use is the single most preventable risk factor for cancer and cancer-related deaths in Japan, as it is in most countries worldwide [5,16,17]. 

Health warnings are an effective strategy for increasing knowledge about smoking-related health harms to smokers and non-smokers, as well as encouraging smokers to quit, and preventing smoking initiation among non-smokers [18,19,20,21,22]. There is strong evidence that pictorial warnings are more effective than text-only warnings [22,23]. A systematic review found that cigarette warnings — especially pictorial warnings—are associated with increased knowledge of smoking-related harms, knowledge of quitlines and quitline calls, increased quit attempts and short-term quitting, and reduced smoking prevalence [24]. A meta-analysis of experimental studies also concluded that pictorial warnings are more effective in capturing attention, increasing thoughts about smoking harms, preventing smoking initiation, and increasing quit intentions than text-only warnings [25]. Similarly, a recent review of studies conducted in Asian countries found that pictorial warnings are more effective for increasing health knowledge, warning salience, and quit intentions, and deterring smoking initiation than text-only warnings [26].

**Figure 1:**
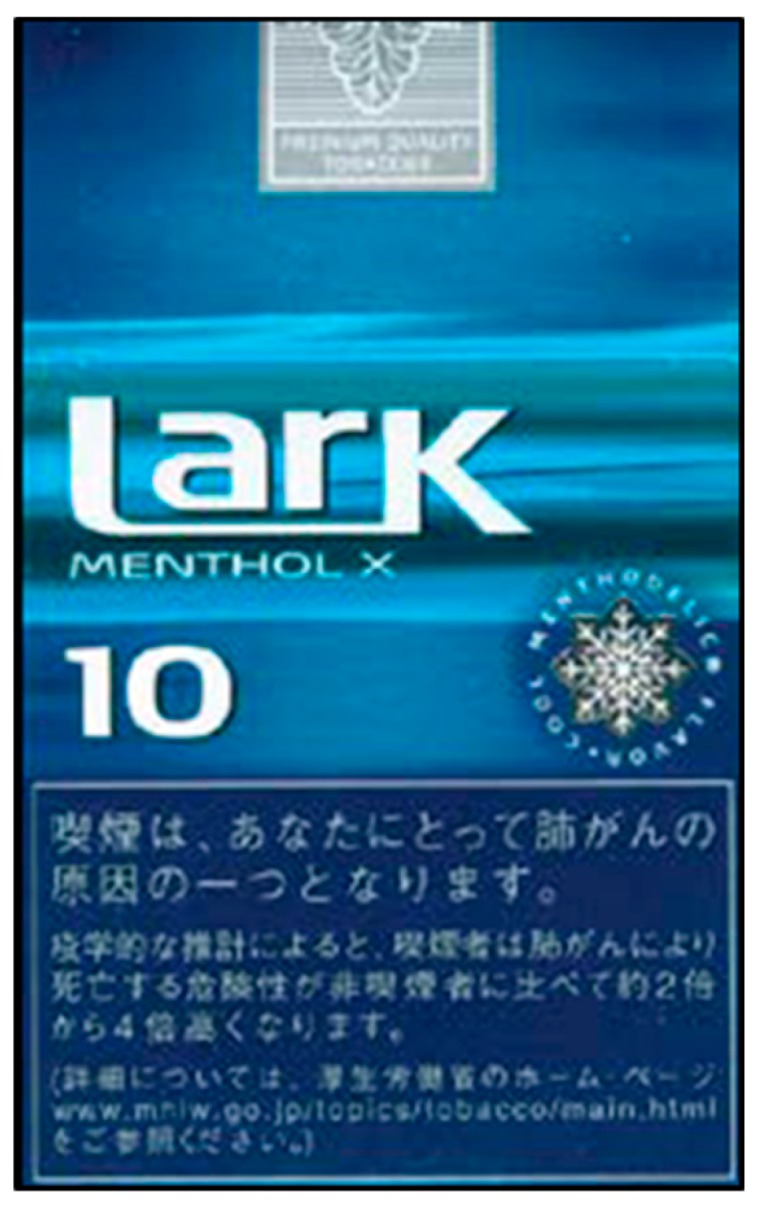
The 2018 Japanese text-only health warning (front of cigarette pack) [28].

Japan became a Party to the World Health Organization Framework Convention on Tobacco Control (WHO FCTC) in February 2005. However, their progress in the implementation of effective tobacco control policies has been slow in comparison with other high-income countries. Since 2005, Japan has required text-only health warnings (two to eight messages in Japanese to be displayed at any given time) that cover 30% of the front and back of cigarette packs, which only meet minimal standards recommended under FCTC Article 11 and its guidelines (see Figure 1). Text-only warnings are also required on 30% of the front and back of packaging for heated tobacco product (HTP) devices that produce a nicotine aerosol for oral inhalation (two messages in Japanese, one on front and one on back of packs). Further details on message content of health warnings on cigarette and HTP packs are presented in Table 1. In December 2018, the Ministry of Finance announced plans to increase the size of text-only health warnings to cover at least 50% of the front and back of both cigarette and HTP packs before the 2020 Summer Olympics and Paralympic Games in Tokyo (Tokyo 2020) [27].

Currently, very little is known about the effectiveness of Japan’s text-only health warnings. One experimental study of adult (≥20 years) smokers in Japan found that pictorial warnings on cigarette packs were perceived as leaving more of an impression, more eye-catching, more frightening, easier to understand, more convincing, and more likely to encourage smokers to share health information with a smoking friend, compared to current text-only warnings [29]. To our knowledge, there have been no population-based studies of the effectiveness of the existing text-only cigarette health warnings in Japan.

In this study, we focus on examining the impact of text-only cigarette health warnings in Japan, as measured by key indicators of warning effectiveness: (1) salience (noticing and reading warnings); (2) behavioral responses (forgoing cigarette because of warnings and avoiding warnings); and (3) cognitive responses (thinking about harms and quitting because of warnings). We also examined the level of public support for pictorial warnings in Japan. 

## 2. Materials and Methods

### 2.1. Data Source and Sample

Data are from Wave 1 of the International Tobacco Control (ITC) Japan Survey, a cohort survey of adult (≥20 years) cigarette smokers, HTP users, cigarette and HTP dual users, and non-users of cigarettes and HTPs (hereinafter referred to as non-users). 

A full description of the study methods (compliant with the Checklist for Reporting Results of Internet E-Surveys [30]) and survey design is available elsewhere [31]. Briefly, respondents were recruited from the Rakuten Insight panel in Japan. The survey was administered via the web in Japanese from February to March 2018 and took approximately 27 min to complete. 

The sample comprised the following cohorts: 1) current cigarette smokers (n = 3306), 2) HTP only users (n = 207), 3) cigarette and HTP dual users (n = 555), and 4) non-users (n = 616). The Rakuten web panel is designed to be nationally representative of the Japanese population. Sampling weights were computed for all respondents and calibrated to target figures from the 2017 Japan Society and New Tobacco Internet Survey [32] to ensure that the final sample was representative of Japanese smokers, HTP only users, cigarette and HTP dual users, and non-users. The survey cooperation and response rates for the total sample were 96.3% and 45.1%, respectively, which are high within the typical range for online surveys [30,33,34]. Research ethics approval was obtained from the Office of Research Ethics at the University of Waterloo, Canada.

The analyses presented here are based on a total sample of 4684 respondents: 3306 current cigarette smokers, 555 dual users (cigarette smokers who also use HTP), and 823 non-cigarette smokers (HTP only users, and non-users of cigarettes and HTP). 

### 2.2. Measures

#### 2.2.1. Demographics

Demographic variables were: region of residence (Hokkaido/Tohoku, Kanto, Chubu/Kansai, Chugoku/Shikoku/Kyushu/Okinawa), age (20–29, 30–39, 40–59, 60+), sex (female, male), education level (low: junior high school/high school, moderate: vocational school/junior college/technical college, high: undergraduate/postgraduate, no answer), and annual household income (low: <4 million yen, moderate: 4 to 6 million yen, high: >6 million yen, no answer).

#### 2.2.2. Smoking Status Classification

Because this study focuses on the effectiveness of cigarette health warnings, smoking status classification was based on whether respondents smoke cigarettes and are exposed to cigarette pack warnings. *Current cigarette smokers* were defined as those who smoked at least 100 cigarettes during their lifetime and smoked cigarettes at least monthly and used HTP less than weekly. *Dual users* were defined as those who smoked cigarettes at least monthly and used HTP at least weekly. *Non-cigarette smokers* were defined as those who: (1) have quit smoking cigarettes, smoked less than monthly or never smoked cigarettes AND reported any current use of HTP (*HTP only users*), or (2) have quit smoking cigarettes, smoked less than monthly, or never smoked cigarettes AND reported no current use of HTP (*non-users of cigarettes and HTPs*). Further details on smoking status classification are provided in Appendix A.

#### 2.2.3. Health Warning Effectiveness

We used six measures that have been identified as key indicators of health warning effectiveness [35]. Previous research evaluating the impact of health warnings have used these measures, including studies conducted by the International Tobacco Control Policy Evaluation Project (ITC Project) in Canada, the United States, the United Kingdom, and Australia [36], Uruguay [37], Brazil [14], Mexico [38], Thailand [39], Malaysia [40], China [41], Mauritius [42], and six European countries (Germany, Greece, Hungary, Poland, Romania, and Spain) [43]. Studies have demonstrated that these key indicators of warning effectiveness are significant predictors of important outcomes, such as subsequent quit intentions and quit attempts [44].

Health warning *salience* among cigarette smokers, dual users, and non-cigarette smokers was measured by two questions: (1) ‘In the last 30 days, how often have you noticed the warning labels on cigarette packages?’, and (2) ‘In the last 30 days, how often have you read or looked closely at warning labels on cigarette packages?’ (for respondents who reported noticing warnings). Among those respondents who noticed, and read or looked closely at warnings, responses were dichotomized as ‘often/very often’ and ‘never/rarely/sometimes’. Responses ‘refused’ and ‘don’t know’ were excluded. 

*Behavioral* reactions among cigarette smokers and dual users were measured by two questions: (1) ‘In the last 30 days, have the warning labels stopped you from having a cigarette when you were about to smoke one?’ Responses were dichotomized as ‘once/a few times/many times’ and ‘never’; responses ‘refused’ and ‘don’t know’ were excluded; and (2) ‘In the last 30 days, have you made any effort to avoid looking at or thinking about the warning labels—such as covering them up, keeping them out of sight, using a cigarette case, avoiding certain warnings, or any other means?’ Responses were ‘yes’ and ‘no’; responses ‘refused’ and ‘don’t know’ were excluded. 

*Cognitive* reactions among cigarette smokers, dual users, and non-cigarette smokers were measured by two questions: (1) ‘To what extent do the warning labels make you think about the health risks of smoking cigarettes?’, and (2) ‘To what extent do the warning labels on cigarette packs make you more likely to quit smoking?’ (for respondents who were cigarette smokers or dual users) Responses were dichotomized as ‘a lot’ and ‘not at all/a little’. Responses ‘refused’ and ‘don’t know’ were excluded. 

#### 2.2.4. Support for Pictorial Health Warnings

Support for pictorial health warnings among cigarette smokers, dual users, and non-cigarette smokers was measured by the question: ‘Would you support or oppose the government including pictures as part of the warning labels on cigarette packs?’ Responses were dichotomized as ‘strongly support/support’ and ‘oppose/strongly oppose/don’t know’. Response ‘refused’ was excluded. 

### 2.3. Data Analysis

Demographic and smoking characteristics of the sample were estimated using unweighted descriptive statistics. Weighted multivariable logistic regression models were used to examine the demographic and behavioral characteristics associated with each of the six indicators of health warning effectiveness, and support for pictorial health warnings. Regression models controlled for sex, age group, household income, education level, and smoking status. All analyses were conducted with SAS version 9.4 software (Research Triangle Institute, Cary, NC, USA).

## 3. Results

### 3.1. Sample Characteristics

Table 2 presents the sample characteristics. Overall, the majority of respondents were male (70.0%) and cigarette smokers (70.6%). Most were aged 40 to 59 years (42.1%) and had high education (45.0%). 

### 3.2. Predictors of Health Warning Effectiveness

Table 3, Table 4, Table 5 and Table 6 present the results of the weighted multiple logistic regression models of predictors for each of the six indicators of health warning effectiveness, controlling for demographic covariates. Differences in the sample size between models are due to the fact that only respondents with complete data for all covariates and outcome measures were included in the analysis. 

### 3.3. Health Warning Salience

Overall, 15.6% of respondents noticed health warnings ‘often/very often’. Cigarette smokers (29.7%) and dual users (20.6%) were more likely to notice health warnings than non-cigarette smokers (11.7%). Females (12.2%) were less likely to notice warnings than males (18.7%). Respondents aged 30–39 years (21.3%) were more likely to notice warnings than respondents aged 20–29 years (10.3%). There were no other differences by age, income, and education (see Table 3).

Overall, 7.9% of respondents read or looked closely at health warnings. Cigarette smokers (13.7%) and dual users (11.3%) were more likely to read or look closely at the warnings than non-cigarette smokers (6.2%). Females (5.6%) were less likely to read or look at warnings than males (9.7%). Respondents with higher income (11.8%) were more likely to read or look closely at health warnings than those with lower income (4.9%). There were no other differences by age and education (see Table 3). 

### 3.4. Behavioral Reactions to Health Warnings

Overall, 10.3% of cigarette smokers and dual users said that health warnings stopped them from having a cigarette when they were about to smoke one. Dual users (17.1%) were more likely to forgo a cigarette because of health warnings than cigarette smokers (9.8%). Younger respondents aged 20 to 29 years (16.7%) were more likely to forgo a cigarette than respondents in all other age groups (8.4 to 11.6%). Respondents with moderate (11.4%) to higher income (11.9%) were more likely to forgo a cigarette than those with lower income (7.9%). There were no other differences by sex and education (see Table 4). 

Overall, 7.2% of cigarette smokers and dual users avoided looking at or thinking about health warnings. Cigarette smokers (6.9%) were less likely to avoid looking at or thinking about warnings than dual users (10.7%). Younger respondents aged 20 to 29 years (12.4%) were more likely to avoid warnings than respondents in all other age groups (6.3 to 7.0%). There were no other differences by sex, income, and education (see Table 4).

### 3.5. Cognitive Reactions to Health Warnings

Overall, 27.5% of respondents said that warnings made them think about the health risks of smoking cigarettes ‘a lot’. Cigarette smokers (9.1%) and dual users (13.1%) were less likely to say that the warnings made them think about health risks of smoking than non-cigarette smokers (32.2%). Older respondents aged 60+ years (35.2%) were more likely to say that health warnings made them think about the health risks of smoking than young respondents aged 20 to 29 years (19.8%). There were no other differences by sex, income, and education (see Table 5).

Overall, only 2.7% of cigarette smokers and dual users said that health warnings made them ‘a lot’ more likely to quit smoking cigarettes, with no differences between cigarette smokers (2.7%) and dual users (3.4%). There were no other differences by sex, age, income, and education (see Table 5).

### 3.6. Support for Pictorial Warnings on Cigarette Packs

Overall, 57.6% of respondents said that they would ‘strongly support/support’ pictorial warnings on cigarette packs. In the weighted multiple regression model of predictors of support for pictorial warnings, lower support was reported by cigarette smokers (29.4%) and dual users (36.3%) compared to non-cigarette smokers (64.2%). Respondents with moderate (67.3%) and higher income (60.4%) were more likely to support pictorial warnings than those with lower income (47.6%). There were no other differences by sex, age, and education (see Table 6).

## 4. Discussion

This study demonstrates that Japanese text-only cigarette pack warnings are largely ineffective. The warnings generally fail to capture people’s attention, including smokers who have the greatest exposure to health warning messages on cigarette packs. For example, only 13.7% of Japanese smokers and 11.3% of dual users read or looked closely at the warnings. About one-quarter of smokers (29.7%) and dual users (20.6%) noticed the warnings—which is much lower than the percentage of smokers who noticed warnings in Australia (52.0%) and China (43.1%) at the time when these two countries had text-only warnings of a similar size as Japan’s current warnings; and in the United Kingdom (44.0%) at the time when they had text-only warnings that were even smaller than Japan’s current warnings [40,45]. The weak salience of Japan’s text-only warnings is even more pronounced when compared to significant increases in smokers’ noticing of warnings following the change from text-only to pictorial warnings in other countries, such as Malaysia (54 to 67%) [40], Thailand (62 to 70%) [46], and Mauritius (58 to 83%) [42].

Behavioral reactions to the Japanese warnings also point to their lack of impact. For example, only 1 in 10 smokers are forgoing cigarettes because of the warnings or making efforts to avoid the warnings, which are behaviors that have been shown to predict quit attempts in population-based studies from many countries, including Australia, Canada, the United Kingdom, the United States, Thailand, and Malaysia [12,13,39,47,48].

The Japanese health warnings seem to be helpful for conveying health information to non-cigarette smokers, by encouraging 32.2% to think about the health risks of smoking. Nevertheless, only 9.1% of smokers and 13.1% of dual users said that health warnings made them think about the health risks of smoking, and 2.7% of smokers and 3.4% of dual users said that the warnings made them more likely to quit. These findings are consistent with previous studies conducted in China and Malaysia, which showed that text-only warnings encouraged less than 9.0% of smokers in both countries to think about smoking-related health risks. Although the percentage of smokers who said that text-only warnings made them more likely to quit was also low in both countries (6.6% in China, 15.0% in Malaysia) [40,49], it was still many times higher than it was among smokers in Japan.

Differences in the effectiveness of health warnings across different countries are likely due to factors that were not assessed in this study. For example, the salience of health warnings and smokers’ behavioral and cognitive responses to warnings may be affected by country-specific factors, such as the strength and comprehensiveness of existing tobacco control programs, and social norms for tobacco use, in addition to individual-level factors, such as health knowledge, level of nicotine dependence, and perceptions of the risks of smoking.

Findings from this study highlight the need for stronger health warnings in Japan. In December 2018, the Japanese Ministry of Finance decided to increase the size of text-only warnings from 30 to 50% of the front and back of cigarette packs, which will come into effect on 1 April 2020, three months prior to Tokyo 2020. While the forthcoming health warnings meet the WHO FCTC recommended warning size of 50% of the front and back of cigarette packs, they still fail to meet WHO FCTC Article 11 guidelines that call for the use of pictorial warnings covering more than 50% of the front and back of packs.

The present study found that the majority of respondents (57.6%) want the Japanese government to implement pictorial warnings on cigarette packs, which is lower than public support in Russia (87.0%) and Brazil (76.0%) [50,51]. Support for pictorial warnings in Japan was highest among non-cigarette smokers (64.2%), followed by dual users (36.3%), and lowest among cigarette smokers (29.4%)—levels well below support previously reported by non-smokers in China (80.2%) [41], as well as smokers in Russia (80.0%) and Brazil (73.0%) [50,51]. Based on previous research showing increased support for smoke-free laws and plain packaging after policy implementation [52,53,54,55,56], it is likely that support for pictorial warnings would increase if the policy were to be implemented in Japan. 

It is unclear why support for pictorial warnings is lower in Japan compared to other countries. One possibility is that the Japanese population—especially smokers—have a poor understanding of the negative health impacts of smoking due to the lack of comprehensive public education programmes. Indeed, the 2017 WHO report on the global epidemic showed that no national mass media campaigns about the dangers of tobacco use and benefits of quitting were conducted in Japan between July 2014 and June 2016 [57]. National campaigns are needed to educate people about the dangers of tobacco use and to build public support for stronger health warnings in Japan. Mass media campaigns that are designed to reinforce health warning messages—such as those conducted by the Australian government when pictorial warnings were first introduced in the country [58]—are likely to enhance the effectiveness of health warnings for communication of the consequences of tobacco use in order to encourage cessation and prevent uptake [59]. Studies showing increases in knowledge for various health effects following the implementation of pictorial health warnings in Australia, Canada, Mexico, Thailand, and the United Kingdom [24] suggest that Japanese health warnings should be strengthened in order to target gaps in smoking-related health knowledge, which may then encourage more smokers to quit [60].

This study has several limitations. First, self-reported measures may be subject to response bias. Second, our survey design did not allow for examination of the effectiveness of individual health warnings. Existing experimental literature demonstrates that pictorial cigarette warnings are more effective than text-only warnings [25], but more experimental research is needed to better understand whether the impact of Japan’s health warnings depends on the specific content, design elements, and other features of warning messages. Third, findings are based on cross-sectional data, and we were not able to compare the impact of text-only versus pictorial warnings. Longitudinal studies will be important to track whether the low effectiveness of Japan’s text-only warnings is maintained. But a more likely outcome over time is that the effectiveness of Japan’s warnings would actually decrease because of the known phenomenon of “warning wear-out”—warnings become less effective over repeated exposures. ITC studies have documented warning wear-out in several countries, including Canada and the United States [61] and Mauritius [42]. Longitudinal studies would also be valuable to measure the likely increase in health warning effectiveness if Japan’s text-only warnings are replaced with pictorial warnings, as called for by the WHO FCTC. Finally, we did not examine the effectiveness of Japan’s health warnings on packaging for HTPs, which are recognized as tobacco products under the WHO FCTC and thus subject to the same provisions for cigarettes [62]. 

## 5. Conclusions

The current findings demonstrate the low effectiveness of text-only warnings in Japan across all key indicators of health warning salience, and behavioral and cognitive responses to the warnings. Japan’s text-only warnings only meet WHO FCTC minimum standards, and are weak compared to large pictorial warnings that have been implemented in other high-income countries such as New Zealand, Hong Kong, and Australia; and low and middle-income countries such as Timor-Leste, Nepal, and India [28]. There is a critical need to implement stronger health warnings in Japan that comply with WHO FCTC Article 11 and its guidelines, which previous studies have found to be important for communicating information on the health risks of smoking to the public, motivating smokers to quit, and reducing smoking initiation.

## Figures and Tables

**Table 1:** Text-only health warning messages on cigarette and Heated Tobacco Product (HTP) packs in Japan, 2005–2018.

	Health Warnings on Cigarette Packs	Health Warnings on HTP Packs
Front of pack (in Japanese)	Smoking is a cause of lung cancer. According to epidemiological estimates, smokers are about two to four times more likely than non-smokers to die of lung cancer.	Smoking is a cause of lung cancer, and can increase the risk of myocardial infarction, stroke, and aggravate the symptoms of emphysema.
	Smoking increases risk of myocardial infarction. According to epidemiological estimates, smokers are about 1.7 times more likely than non-smokers to die of a heart attack.	
	Smoking increases risk of stroke. According to epidemiological estimates, smokers are about 1.7 times more likely than non-smokers to die of a stroke.	
	Smoking can aggravate the symptoms of emphysema.	
Back of pack (in Japanese)	Smoking during pregnancy is a cause of preterm delivery and impaired fetal growth. According to epidemiological estimates, pregnant women who smoke have almost double the risk of low birth weight and three times the risk of premature birth than pregnant women who do not smoke.	The degree may differ from person to person, but nicotine causes addiction to smoking. Smoking while underage heightens the addiction and damage to health caused by cigarettes. Never smoke, even if encouraged to by those around you.
	Tobacco smoke adversely affects the health of people around you, especially infants, children and the elderly. When smoking, be careful not to inconvenience others.	
	The degree may differ from person to person, but nicotine [in cigarettes] causes addiction to smoking.	
	Smoking while underage heightens the addiction and damage to health caused by cigarettes. Never smoke, even if encouraged to by those around you.	

**Table 2:** Demographic and smoking characteristics of sample.

Characteristic	Unweighted Frequency (%) of Respondents (n = 4684)
Sex	
Female	1399 (30.0%)
Male	3285 (70.0%)
Age	
20–29	479 (10.2%)
30–39	981 (21.0%)
40–59	1973 (42.1%)
60+	1251 (26.7%)
Income	
Low	1223 (26.1%)
Moderate	1059 (22.6%)
High	1806 (38.6%)
No answer	596 (12.7%)
Education	
Low	1672 (35.7%)
Moderate	850 (18.1%)
High	2107 (45.0%)
No answer	55 (1.2%)
Smoking status	
Cigarette smokers	3306 (70.6%)
Dual users of cigarettes and HTP	555 (11.6%)
Non-cigarette smokers	823 (17.6%)

**Table 3:** Factors associated with whether cigarette smokers, dual users, and non-cigarette smokers noticed health warnings (N = 4563) and read or looked closely at health warnings (n = 3892).

	Noticed Health Warnings ‘Often/Very Often’ n = 1316		Read or Looked Closely at Health Warnings ‘Often/Very Often’ n = 642	
	% (95% CI)	aOR (95% CI)	% (95% CI)	aOR (95% CI)
Overall	15.6 (13.3–18.3)	--	7.9 (6.3–9.9)	--
Sex				
Male	18.7 (14.5–23.7)	1.00	9.7 (7.0–13.4)	1.00
Female	12.2 (9.6–15.4)	0.59 (0.37–0.92) *	5.6 (3.9–7.9)	0.54 (0.31–0.94) *
Age				
20–29	10.3 (6.1–16.7)	1.00	5.8 (2.8–11.6)	1.00
30–39	21.3 (14.1–30.8)	2.51 (1.11–5.68) *	9.9 (5.9–16.3)	1.84 (0.68–5.02)
40–59	16.9 (12.8–22.1)	1.85 (0.91–3.75)	7.3 (4.9–10.6)	1.29 (0.51–3.23)
60+	14.2 (11.0–18.2)	1.48 (0.76–2.89)	8.6 (5.9–12.2)	1.55 (0.63–3.81)
Income				
Low	14.9 (10.8–20.2)	1.00	4.9 (3.3–7.3)	1.00
Moderate	15.1 (10.9–20.5)	1.02 (0.58–1.78)	5.6 (3.5–8.8)	1.15 (0.60–2.20)
High	17.2 (13.3–21.9)	1.20 (0.72–2.00)	11.8 (8.5–16.2)	2.69 (1.52–4.74) ***
No answer	13.5 (9.0–19.7)	0.88 (0.47–1.65)	6.9 (3.9–11.9)	1.44 (0.68–3.06)
Education				
Low	15.2 (11.4–20.0)	1.00	9.2 (5.9–14.1)	1.00
Moderate	15.2 (10.6–21.3)	1.00 (0.57–1.73)	6.1 (4.0–9.3)	0.63 (0.33–1.21)
High	15.8 (12.6–19.6)	1.05 (0.67–1.66)	8.0 (5.9–10.8)	0.85 (0.45–1.60)
No answer	31.6 (9.0–68.4)	2.83 (0.50–16.09)	7.2 (2.7–17.8)	0.75 (0.24–2.40)
Smoking status				
Non-cigarette smokers	11.7 (8.8–15.5)	1.00	6.2 (4.3–9.0)	1.00
Cigarette smokers	29.7 (26.8–32.8)	3.28 (2.26–4.77) ***	13.7 (11.8–15.9)	2.47 (1.55–3.94) ***
Dual users	20.6 (16.3–25.7)	1.98 (1.23–3.19) **	11.3 (8.3–15.2)	1.96 (1.09–3.51) *

Significance levels are indicated as follows: * *p* < 0.05; ** *p* < 0.01; *** *p* < 0.001. CI, confidence interval; aOR, adjusted odds ratio of noticing warnings (0: never/rarely/sometimes, 1: often/very often); reading or looking at warnings (0: never/rarely/sometimes, 1: often/very often).

**Table 4:** Factors associated with whether cigarette smokers and dual users said health warnings stopped them from having a cigarette when they were about to smoke one (n = 3761) and whether they avoided looking at or thinking about the health warnings in the last 30 days (n = 3701).

	Health Warnings Stopped You from Having a Cigarette at Least Once n = 404		Avoided Looking at or Thinking about Health Warnings n = 276	
	% (95% CI)	aOR (95% CI)	% (95% CI)	aOR (95% CI)
Overall	10.3 (9.3–11.5)	--	7.2 (6.3–8.2)	--
Sex				
Male	9.7 (8.6–10.9)	1.00	7.0 (6.0–8.1)	1.00
Female	12.0 (9.5–14.9)	1.27 (0.94–1.72)	7.7 (5.8–10.1)	1.11 (0.78–1.58)
Age				
20–29	16.7 (12.4–22.2)	1.00	12.4 (9.1–16.7)	1.00
30–39	11.6 (9.3–14.3)	0.65 (0.43–0.99) *	7.0 (5.3–9.2)	0.53 (0.34–0.84) **
40–59	9.3 (7.9–11.0)	0.51 (0.34–0.76) ***	6.5 (5.2–8.1)	0.49 (0.32–0.74) ***
60+	8.4 (6.6–10.7)	0.45 (0.29–0.70) ***	6.3 (4.7–8.2)	0.47 (0.30–0.74) **
Income				
Low	7.9 (6.2–9.9)	1.00	6.6 (5.0–8.6)	1.00
Moderate	11.4 (9.1–14.3)	1.52 (1.06–2.18) *	6.7 (5.1–8.7)	1.01 (0.67–1.54)
High	11.9 (10.1–13.9)	1.59 (1.15–2.19) **	8.9 (7.3–10.7)	1.39 (0.96–2.01)
No answer	9.1 (6.0–13.4)	1.17 (0.69–1.97)	4.5 (2.8–7.1)	0.66 (0.37–1.19)
Education				
Low	9.0 (7.4–10.8)	1.00	7.5 (6.0–9.4)	1.00
Moderate	10.5 (8.1–13.7)	1.20 (0.83–1.73)	6.4 (4.5–8.9)	0.83 (0.53–1.30)
High	11.0 (9.3–12.8)	1.25 (0.94–1.66)	7.4 (6.1–8.9)	0.98 (0.71–1.34)
No answer	18.9 (6.9–42.3)	2.40 (0.72–7.95)	3.0 (0.6–14.6)	0.38 (0.07–2.14)
Smoking status				
Non-cigarette smokers	--	--	--	--
Cigarette smokers	9.8 (8.7–11.1)	1.00	6.9 (6.0–8.0)	1.00
Dual users	17.1 (13.8–21.0)	1.91 (1.43–2.54) ***	10.7 (8.1–14.1)	1.62 (1.15–2.30) **

Significance levels are indicated as follows: * *p* < 0.05; ** *p* < 0.01; *** *p* < 0.001. CI, confidence interval; aOR, adjusted odds ratio of stopping from having cigarette (0: never, 1: once/a few times/many times); avoiding or thinking about warnings (0: no, 1: yes).

**Table 5:** Factors associated with whether cigarette smokers, dual users, and non-cigarette smokers said health warnings made them think about the risks of smoking (N = 4404) and made them more likely to quit smoking (n = 3602).

	Health Warnings Made You Think about the Risks of Smoking Cigarettes ‘a lot’ n = 546		Health Warnings Make You ‘a lot’ More Likely to Quit Smoking n = 100	
	% (95% CI)	aOR (95% CI)	% (95% CI)	aOR (95% CI)
Overall	27.5 (24.0–31.5)	--	2.7 (2.1–3.4)	--
Sex				
Male	27.2 (21.5-33.8)	1.00	2.5 (1.9-3.3)	1.00
Female	27.9 (23.5-32.6)	0.97 (0.64-1.47)	3.4 (2.1-5.5)	0.73 (0.40-1.35)
Age				
20–29	19.8 (11.3–32.4)	1.00	3.2 (1.7–5.9)	1.00
30–39	29.0 (19.6–40.6)	0.59 (0.24–1.43)	3.3 (2.2–5.0)	0.96 (0.46–2.02)
40–59	23.0 (17.5–29.6)	0.82 (0.38–1.77)	2.5 (1.7–3.6)	1.30 (0.62–2.71)
60+	35.2 (29.7–41.2)	0.44 (0.21–0.91) *	2.5 (1.6–3.9)	1.30 (0.59–2.89)
Income				
Low	26.6 (20.4–33.8)	1.00	1.9 (1.1–3.0)	1.00
Moderate	25.9 (18.7–34.6)	1.04 (0.58–1.87)	2.6 (1.6–4.3)	0.70 (0.34–1.45)
High	29.7 (24.1–36.0)	0.85 (0.52–1.37)	3.2 (2.3–4.4)	0.57 (0.31–1.06)
No answer	26.2 (17.4–37.4)	1.02 (0.52–1.99)	3.6 (2.0–6.5)	0.50 (0.22–1.14)
Education				
Low	25.2 (19.6–31.8)	1.00	3.0 (2.1–4.2)	1.00
Moderate	28.9 (21.6–37.5)	0.82 (0.48–1.40)	1.7 (0.9–3.1)	1.80 (0.87–3.72)
High	28.4 (23.3–34.2)	0.84 (0.53–1.32)	3.1 (2.2–4.4)	0.96 (0.57–1.61)
No answer	12.0 (1.9–48.5)	2.57 (0.34–19.56)	--	--
Smoking status				
Non-cigarette smokers	32.2 (27.6–37.1)	1.00	--	--
Cigarette smokers	9.1 (7.8–10.7)	4.85 (3.60–6.55) ***	2.7 (2.1–3.4)	1.00
Dual users	13.1 (9.4–18.0)	3.21 (2.01–5.12) ***	3.4 (2.1–5.4)	0.79 (0.46–1.36)

Significance levels are indicated as follows: * *p* < 0.05; ** *p* < 0.01; *** *p* < 0.001. CI, confidence interval; aOR, adjusted odds ratio of thinking about risks of smoking (0: not at all/a little, 1: a lot); making you more likely to quit (0: not at all/a little, 1: a lot).

**Table 6:** Factors associated with support for pictorial warnings on cigarette packs (N = 4616)**.**

‘Support/Strongly Support’ Pictorial Warnings on Cigarette Packs n = 1628		
	% (95% CI)	aOR (95% CI)
Overall	57.6 (53.8–61.2)	--
Sex		
Male	58.4 (52.1–64.4)	1.00
Female	56.8 (52.1–61.3)	0.93 (0.64–1.34)
Age		
20–29	58.3 (45.8–69.9)	1.00
30–39	57.9 (47.5–67.7)	0.98 (0.47–2.04)
40–59	55.6 (49.3–61.7)	0.88 (0.47–1.66)
60+	59.0 (53.7–64.0)	1.03 (0.56–1.90)
Income		
Low	47.6 (40.4–54.9)	1.00
Moderate	67.3 (59.9–74.0)	2.44 (1.50–3.95) ***
High	60.4 (54.3–66.2)	1.75 (1.14–2.69) *
No answer	55.3 (44.8–65.4)	1.40 (0.81–2.42)
Education		
Low	55.3 (48.3–62.0)	1.00
Moderate	59.5 (52.0–66.5)	1.21 (0.79–1.87)
High	57.8 (52.1–63.3)	1.12 (0.74–1.70)
No answer	61.8 (35.7–82.5)	1.35 (0.40–4.57)
Smoking status		
Non-cigarette smokers	64.2 (59.4–68.7)	1.00
Cigarette smokers	29.4 (26.9–32.1)	0.22 (0.17–0.29) ***
Dual users	36.3 (30.6–42.4)	0.31 (0.21–0.45) ***

Significance levels are indicated as follows: * *p* < 0.05; *** *p* < 0.001; CI, confidence interval; aOR, adjusted odds of support for pictorial warnings (0: oppose/strongly oppose/don’t know, 1: strongly support/support).

## Appendix A. Smoking status classification

In this study, we defined cigarette smokers as those who smoked at least monthly and had smoked at least 100 cigarettes in lifetime, which are well-established criteria used in tobacco surveillance surveys to define smokers who smoke on a regular basis. Given that the majority of monthly HTP users are likely experimenting with these products, we defined HTP users as those who used HTP products at least weekly in order to provide a more accurate description of those who use HTP products on a regular basis. Weighted multivariable logistic regression analyses conducted using the same criterion (i.e., at least weekly use) to define both the cigarette smoker group and the HTP user group did not substantively alter the findings reported in this study.

## References

[1] Zheng W., McLerran D.F., Rolland B.A., Fu Z., Boffetta P., He J., Gupta P.C., Ramadas K., Tsugane S., Irie F. (2014). Burden of Total and Cause-Specific Mortality Related to Tobacco Smoking among Adults Aged ≥ 45 Years in Asia: A Pooled Analysis of 21 Cohorts. PLoS Med..

[2] Drope J., Schluger N., Cahn Z., Drope J., Hamill S., Islami F., Liber A., Nargis N., Stoklosa M. (2018). The Tobacco Atlas.

[3] GBD 2015 Tobacco Collaborators (2017). Tobacco Collaborators Smoking prevalence and attributable disease burden in 195 countries and territories, 1990–2015: A systematic analysis from the Global Burden of Disease Study 2015. Lancet.

[4] Katanoda K., Marugame T., Saika K., Satoh H., Tajima K., Suzuki T., Tamakoshi A., Tsugane S., Sobue T. (2008). Population Attributable Fraction of Mortality Associated with Tobacco Smoking in Japan: A Pooled Analysis of Three Large-scale Cohort Studies. J. Epidemiol..

[5] Ikeda N., Inoue M., Iso H., Ikeda S., Satoh T., Noda M., Mizoue T., Imano H., Saito E., Katanoda K. (2012). Adult Mortality Attributable to Preventable Risk Factors for Non-Communicable Diseases and Injuries in Japan: A Comparative Risk Assessment. PLoS Med..

[6] Murakami Y., Miura K., Okamura T., Ueshima H. (2011). Population attributable numbers and fractions of deaths due to smoking: A pooled analysis of 180,000 Japanese. Prev. Med..

[7] Gu D., Kelly T.N., Wu X., Chen J., Samet J.M., Huang J.-F., Zhu M., Chen J.-C., Chen C.-S., Duan X. (2009). Mortality Attributable to Smoking in China. N. Engl. J. Med..

[8] Yang J.J., Yu D., Wen W., Shu X.-O., Saito E., Rahman S., Gupta P.C., He J., Tsugane S., Xiang Y.-B. (2019). Tobacco Smoking and Mortality in Asia: A Pooled Meta-analysis. JAMA Netw. Open.

[9] Sakata R., McGale P., Grant E.J., Ozasa K., Peto R., Darby S.C. (2012). Impact of smoking on mortality and life expectancy in Japanese smokers: A prospective cohort study. BMJ.

[10] Ikeda N., Saito E., Kondo N., Inoue M., Ikeda S., Satoh T., Wada K., Stickley A., Katanoda K., Mizoue T. (2011). What has made the population of Japan healthy?. Lancet.

[11] Yamaguchi T., Nishiura H., Yamaguchi T., Nishiura H. (2019). Predicting the epidemiological dynamics of lung cancer in Japan. J. Clin. Med..

[12] Borland R., Yong H.-H., Wilson N., Fong G.T., Hammond D., Cummings K.M., Hosking W., McNeill A. (2009). How reactions to cigarette packet health warnings influence quitting: Findings from the ITC Four-Country survey. Addiction.

[13] Fathelrahman A.I., Omar M., Awang R., Borland R., Fong G.T., Hammond D., Zain Z. (2009). Smokers’ responses toward cigarette pack warning labels in predicting quit intention, stage of change, and self-efficacy. Nicotine Tob. Res..

[14] Thrasher J.F., Villalobos V., Szklo A., Fong G.T., Pérez C., Sebrié E., Sansone N., Figueiredo V., Boado M., Arillo-Santillán E. (2010). Assessing the impact of cigarette package health warning labels: A cross-country comparison in Brazil, Uruguay and Mexico. Salud Pública De México.

[15] Inoue M., Iwasaki M., Otani T., Sasazuki S., Tsugane S. (2006). Public awareness of risk factors for cancer among the Japanese general population: A population-based survey. BMC Public Health.

[16] GBD 2015 Tobacco Collaborators (2016). Risk Factors Collaborators Global, regional, and national comparative risk assessment of 79 behavioural, environmental and occupational, and metabolic risks or clusters of risks, 1990–2015: A systematic analysis for the Global Burden of Disease Study 2015. Lancet.

[17] GBD 2016 Tobacco Collaborators (2017). Risk Factors Collaborators Global, regional, and national comparative risk assessment of 84 behavioural, environmental and occupational, and metabolic risks or clusters of risks, 1990–2016: A systematic analysis for the Global Burden of Disease Study 2016. Lancet.

[18] Hammond D., Fong G.T., McNeill A., Borland R., Cummings K.M. (2006). Effectiveness of cigarette warning labels in informing smokers about the risks of smoking: Findings from the International Tobacco Control (ITC) Four Country Survey. Tob. Control..

[19] Hammond D., Fong G.T., McDonald P.W., Cameron R., Brown K.S. (2003). Impact of the graphic Canadian warning labels on adult smoking behaviour. Tob. Control..

[20] Azagba S., Sharaf M.F. (2013). The effect of graphic cigarette warning labels on smoking behavior: Evidence from the Canadian experience. Nicotine Tob. Res..

[21] Shanahan P., Elliott D. (2009). Evaluation of the Effectiveness of Graphic Health Warnings on Tobacco Product Packaging 2008—Executive Summary.

[22] Hammond D. (2011). Health warning messages on tobacco products: A review. Tob. Control..

[23] Fong G.T., Hammond D., Hitchman S.C. (2009). The impact of pictures on the effectiveness of tobacco warnings. Bull. World Health Organ..

[24] Noar S.M., Francis D.B., Bridges C., Sontag J.M., Ribisl K.M., Brewer N.T. (2016). The impact of strengthening cigarette pack warnings: Systematic review of longitudinal observational studies. Soc. Sci. Med..

[25] Noar S.M., Hall M.G., Francis D.B., Ribisl K.M., Pepper J.K., Brewer N.T. (2016). Pictorial cigarette pack warnings: A meta-analysis of experimental studies. Tob. Control..

[26] Ratih S.P., Susanna D. (2018). Perceived effectiveness of pictorial health warnings on changes in smoking behaviour in Asia: A literature review. BMC Public Health.

[27] Japanese Cigarette Packs May Get Bigger Health Warning Label. https://www.japantimes.co.jp/news/2016/02/01/national/japanese-cigarette-packs-may-get-bigger-health-warning-label/#.Xjg1QGhKiUk.

[28] Canadian Cancer Society (2018). Cigarette Package Health Warnings.

[29] Chéron E. (2015). Effect of Graphic Images in Cigarette Health Warning: A Call for Stricter Packaging Regulation in Japan. J. Int. Consum. Mark..

[30] Eysenbach G., Schonlau M. (2004). Improving the Quality of Web Surveys: The Checklist for Reporting Results of Internet E-Surveys (CHERRIES). J. Med. Internet Res..

[31] ITC Project ITC Japan Survey Wave 1 and 1.5 Technical Report. https://itcproject.org/methods/technical-reports/itc-japan-survey-technical-report-wave-1-and-15-2018-june-2019/.

[32] Tabuchi T., Shinozaki T., Kunugita N., Nakamura M., Tsuji I. (2018). Study profile: The Japan “Society and New Tobacco” Internet Survey (JASTIS): A longitudinal internet cohort study of heat-not-burn tobacco products, electronic cigarettes, and conventional tobacco products in Japan. J. Epidemiol..

[33] Eysenbach G., Wyatt J., McKenzie B. (2002). Using the Internet for Surveys and Health Research. J. Med. Internet Res..

[34] Thompson M.E., Fong G.T., Boudreau C., Driezen P., Li G., Gravely S., Cummings K.M., Heckman B.W., O’Connor R., Thrasher J.F. (2019). Methods of the ITC Four Country Smoking and Vaping Survey, Wave 1 (2016). Addiction.

[35] International Agency for Research on Cancer (2008). IARC Handbooks for Cancer Prevention, Tobacco Control, Volume 12: Methods for Evaluating Tobacco Control Policies.

[36] Borland R., Wilson N., Fong G.T., Hammond D., Cummings K.M., Yong H.-H., Hosking W., Hastings G., Thrasher J., McNeill A. (2009). Impact of graphic and text warnings on cigarette packs: Findings from four countries over five years. Tob. Control..

[37] Gravely S., Fong G.T., Driezen P., McNally M., Thrasher J.F., Thompson M.E., Boado M., Bianco E., Borland R., Hammond D. (2014). The impact of the 2009/2010 enhancement of cigarette health warning labels in Uruguay: Longitudinal findings from the International Tobacco Control (ITC) Uruguay Survey. Tob. Control..

[38] Thrasher J.F., Hammond D., Fong G.T., Arillo-Santillán E. (2007). Smokers’ reactions to cigarette package warnings with graphic imagery and with only text: A comparison between Mexico and Canada. Salud Pública De México.

[39] Li L., Fathelrahman A.I., Borland R., Omar M., Fong G.T., Quah A.C.K., Sirirassamee B., Yong H.-H. (2016). Impact of graphic pack warnings on adult smokers’ quitting activities: Findings from the ITC Southeast Asia Survey (2005–2014). J. Smok. Cessat..

[40] Elton-Marshall T., Xu S.S., Meng G., Quah A.C.K., Sansone G.C., Feng G., Jiang Y., Driezen P., Omar M., Awang R. (2015). The lower effectiveness of text-only health warnings in China compared to pictorial health warnings in Malaysia. Tob. Control..

[41] Li Z., Elton-Marshall T., Fong G.T., Quah A.C.K., Feng G., Jiang Y., Hitchman S.C. (2017). Noticing cigarette health warnings and support for new health warnings among non-smokers in China: Findings from the International Tobacco Control project (ITC) China survey. BMC Public Health.

[42] Green A.C., Kaai S.C., Fong G.T., Driezen P., Quah A.C.K., Burhoo P. (2014). Investigating the effectiveness of pictorial health warnings in Mauritius: Findings from the ITC Mauritius survey. Nicotine Tob. Res..

[43] Trofor A.C., Papadakis S., Lotrean L.M., Radu-Loghin C., Eremia M., Mihaltan F., Driezen P., Kyriakos C.N., Mons U., Demjén T. (2018). Knowledge of the health risks of smoking and impact of cigarette warning labels among tobacco users in six European countries: Findings from the EUREST-PLUS ITC Europe Surveys. Tob. Induc. Dis..

[44] Yong H.-H., Borland R., Thrasher J.F., Thompson M.E., Nagelhout G.E., Fong G.T., Hammond D., Cummings K.M. (2014). Mediational pathways of the impact of cigarette warning labels on quit attempts. Health Psychol..

[45] Hammond D., Fong G.T., Borland R., Cummings K.M., McNeill A., Driezen P. (2007). Text and graphic warnings on cigarette packages. Am. J. Prev. Med..

[46] Yong H.-H., Fong G.T., Driezen P., Borland R., Quah A.C.K., Sirirassamee B., Hamann S., Omar M. (2013). Adult smokers’ reactions to pictorial health warning labels on cigarette packs in Thailand and moderating effects of type of cigarette smoked: Findings from the international tobacco control southeast Asia survey. Nicotine Tob. Res..

[47] Hammond D., Fong G.T., McDonald P.W., Brown K.S., Cameron R. (2004). Graphic Canadian Cigarette Warning Labels and Adverse Outcomes: Evidence from Canadian Smokers. Am. J. Public Health.

[48] Partos T.R., Borland R., Thrasher J.F., Li L., Yong H.-H., O’Connor R.J., Siahpush M. (2014). The predictive utility of micro indicators of concern about smoking: Findings from the International Tobacco Control Four Country study. Addict. Behav..

[49] Fathelrahman A.I., Omar M., Awang R., Cummings K.M., Borland R., Samin A.S.B.M. (2010). Impact of the New Malaysian Cigarette Pack Warnings on Smokers’ Awareness of Health Risks and Interest in Quitting Smoking. Int. J. Environ. Res. Public Health.

[50] Wade B., Merrill R.M., Lindsay G.B. (2011). Cigarette pack warning labels in Russia: How graphic should they be?. Eur. J. Public Health.

[51] Cavalcante T., World Health Organization (2003). Labelling and packaging in Brazil.

[52] Fong G.T., Hyland A., Borland R., Hammond D., Hastings G., McNeill A., Anderson S., Cummings K.M., Allwright S., Mulcahy M. (2006). Reductions in tobacco smoke pollution and increases in support for smoke-free public places following the implementation of comprehensive smoke-free workplace legislation in the Republic of Ireland: Findings from the ITC Ireland/UK Survey. Tob. Control..

[53] Nagelhout G.E., Mons U., Allwright S., Guignard R., Beck F., Fong G.T., de Vries H., Willemsen M.C. (2011). Prevalence and predictors of smoking in “smoke-free” bars. Findings from the International Tobacco Control (ITC) Europe Surveys. Soc. Sci. Med..

[54] Nagelhout G.E., De Vries H., Boudreau C., Allwright S., McNeill A., Putte B.V.D., Fong G.T., Willemsen M.C. (2012). Comparative impact of smoke-free legislation on smoking cessation in three European countries. Eur. J. Public Health.

[55] Wakefield A.M., Hayes L., Durkin S., Borland R. (2013). Introduction effects of the Australian plain packaging policy on adult smokers: A cross-sectional study. BMJ Open.

[56] Swift E., Borland R., Cummings K.M., Fong G.T., McNeill A., Hammond D., Thrasher J.F., Partos T.R., Yong H.-H. (2015). Australian smokers’ support for plain or standardised packs before and after implementation: Findings from the ITC Four Country Survey. Tob. Control..

[57] World Health Organization (2017). WHO Report on the Global Tobacco Epidemic 2017. Country Profile: Japan.

[58] Miller C.L., Hill D.J., Quester P.G., Hiller J.E. (2009). Response of mass media, tobacco industry and smokers to the introduction of graphic cigarette pack warnings in Australia. Eur. J. Public Health.

[59] Hammond D., Wakefield M., Durkin S., Brennan E. (2013). Tobacco packaging and mass media campaigns: Research needs for Articles 11 and 12 of the WHO Framework Convention on Tobacco Control. Nicotine Tob. Res..

[60] Hyland A., Li Q., Bauer J.E., Giovino G.A., Steger C., Cummings K.M. (2004). Predictors of cessation in a cohort of current and former smokers followed over 13 years. Nicotine Tob. Res..

[61] Hitchman S.C., Driezen P., Logel C., Hammond D., Fong G.T. (2014). Changes in effectiveness of cigarette health warnings over time in Canada and the United States, 2002–2011. Nicotine Tob. Res..

[62] Conference of the Parties to the WHO Framework Convention on Tobacco Control Decision FCTC/COP8(22) Novel and Emerging Tobacco Products. https://www.who.int/fctc/cop/sessions/cop8/FCTC_COP8(22).pdf.

